# Assessing the applicability of PMOD residence times model for PET image-based radiation dosimetry

**DOI:** 10.1038/s41598-023-46822-5

**Published:** 2023-11-08

**Authors:** Se Jong Oh, Chul Hyoung Lyoo, Young Hoon Ryu, Jae Yong Choi

**Affiliations:** 1 Division of Applied RI, Korea Institute of Radiological and Medical Sciences, 75 Nowon-ro, Nowon-gu, Seoul, Korea; 2 Department of Neurology, Gangnam Severance Hospital, Yonsei University College of Medicine, Seoul, Korea; 3 Department of Nuclear Medicine, Gangnam Severance Hospital, Yonsei University College of Medicine, Seoul, Korea; 4 Radiological and Medico-Oncological Sciences, University of Science and Technology (UST), Seoul, Korea

**Keywords:** Molecular medicine, Software

## Abstract

The effective dose represents the overall internal radiation exposure to the whole body when exposed to radiation sources. This study aims to compare conventional and software-aided methods to derive the effective dose. In the present study, ^8^F-T807 and ^18^F-Mefway, specific radiotracers for the paired helical tau and serotonin 1A receptor, were administered to healthy subjects (n = 6, each radiotracer), following which whole-body positron emission tomography (PET) images were obtained for 2 h. Subsequently, time-activity curves for major organs were obtained, and the residence times were calculated using the “conventional” and “Residence Times model” tools in PMOD software. The residence times from each method was input into OLINDA/EXM software, and the effective dose was estimated. The differences in the average residence times of the brain, heart, lung, and liver were 18.4, 20.8, 10.4, and 13.3% for ^18^F-T807, and 17.5, 16.4, 18.1, and 17.5% for ^18^F-Mefway, respectively. For the mean effective dose, the error rates between the methods were 3.8 and 1.9% for ^18^F-T807 and ^18^F-Mefway, respectively. The organs that showed the greatest difference in the absorbed dose were the urinary bladder for ^18^F-T807 (40.4%) and the liver for ^18^F-Mefway (14.1%). This method of obtaining the residence time using PMOD can be easily used to derive the effective dose, and is applicable in evaluating the safety of radiotracers for clinical trials.

## Introduction

Positron emission tomography (PET) is a molecular imaging technique that allows the visualization and quantification of biochemical processes in living organisms^1^. Its clinical applications are diverse, ranging from the diagnosis of oncological or neurological diseases to monitoring disease progression and the therapeutic efficacy of certain drugs^2,3^. Overexposure to ionizing radiation poses a potential risk to patients^4^. According to the Code of Federal Regulations in the USA, the effective dose to the whole body should not exceed 30 mSv/administration or 50 mSv/year^5^. Therefore, it is essential to assess the radiation exposure to the whole body to ensure the subject's safety from radiation sources in clinical trials. A common method for assessing radiation exposure is internal dosimetry, which involves calculating the absorbed and effective doses for organs. The absorbed dose quantifies the energy deposited per unit mass from ionizing radiation in materials. Additionally, to determine the relative biological effectiveness of emitted radiation and the differential sensitivity of organs to radiation-induced stochastic effects, the equivalent dose and effective dose are widely considered^6^.

**Figure 1:**
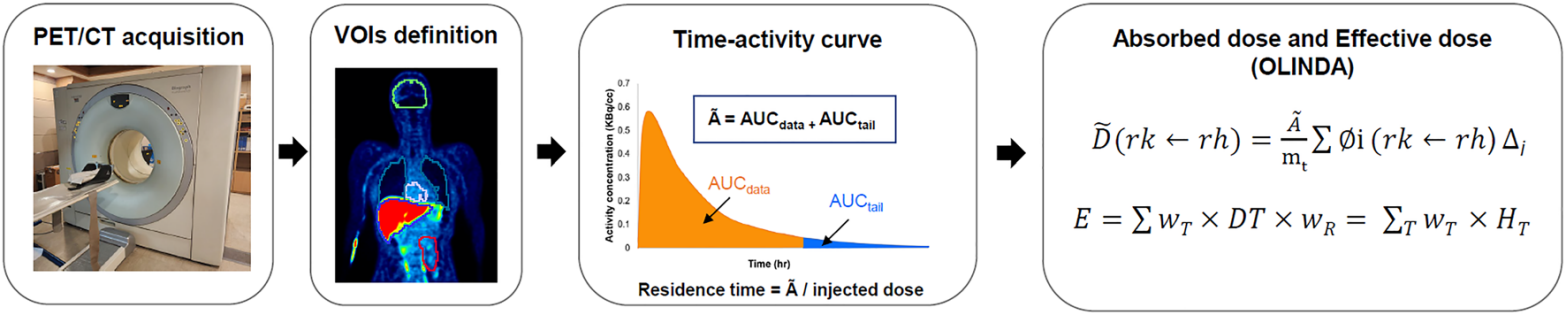
Flowchart for the calculation of the effective dose with the conventional method.

Imaging-based dosimetry is a highly reliable methodology that is frequently used to assess radiation safety during the development of radiotracers^7^. As illustrated Fig. 1, conventional dosimetry involves the acquisition of PET images, determination of the residence time, estimation of the absorbed dose, and calculation of the effective dose^8^. Among these steps, the calculation of the residence time is a time-consuming task that involves the following processes: (i) estimation of the radiation exposure, that is, obtaining the time-activity curves (TACs) for major source organs; ii) calculation of the cumulative activities ($\widetilde{A}$) of the source organs by the area under the curve (AUC); iii) division of the cumulative activity by the injected dose to obtain the residence time. To obtain the cumulative activity, the TAC is integrated using a numerical method. This method involves dividing the AUC into small, finite intervals and estimating the area of each interval using rectangles or trapezoids within the imaging time windows. Thereafter, the AUC from the final acquisition time point to infinity is calculated by integration using a decay function that reflects the physical half-life of the specific isotope, and then added to the cumulative activity obtained from the TAC in the regions of interest.

PMOD is a software that analyzes images acquired from various molecular imaging modalities, such as PET, computed tomography, and magnetic resonance imaging. Furthermore, it offers user-friendly interfaces for organ segmentation, facilitating the application of kinetic modeling; hence, it is widely used by researchers to interpret imaging data. Recently, PMOD Technologies developed a Residence Times model in PKIN that performs dosimetry by automating the calculations for the AUC and residence time with a user-friendly interface. Some researchers have used this model to perform dosimetric studies on humans. Ikawa et. al., used the Residence Times model of PMOD to calculate the effective dose of ^11^C-ER-176 for adults, which is a selective PET tracer that targets the translocator protein 18 kDa. O’Doherty’s groups also used it for ^18^F-tetrafluoroborate, a specific PET tracer for diagnosing thyroid cancer by targeting the human sodium/iodide symporter^9,10^. However, to date, there has been no research to evaluate the effectiveness of PMOD for estimating the residence time compared with the conventional method. Therefore, the present study aimed to evaluate the quantitative accuracy of the Residence Times model of PMOD by comparing the obtained results with those from conventional methods.

## Methods

### Radiotracers

Two different types of radiotracers were used in this study. ^18^F-T807 is a specific radiotracer for paired helical tau, and is used to evaluate Alzheimer’s disease. ^18^F-Mefway is a radiotracer of the serotonin 1A receptor, which is an imaging biomarker of the serotonergic system.

### Study protocol

To measure the radiation doses of the two PET tracers, we recruited 12 volunteers (3 males and 3 females, that is, 6 subjects for each tracer; Table 1). None of the participants had any medical or neuropsychiatric illness history, and the use of PET tracers in human subjects was approved by the Institutional Review Board. This study has been approved by the Institutional Review Board and the Radioactive Drug Research Committee at the Yonsei University College of Medicine. All subjects gave their informed consent prior to their inclusion in the present study. Individual PET data in the present used in previous clinical dosimetry studies^11,12^. These studies were performed in accordance with the ethical standards laid down in the 1964 Declaration of Helsinki and its later amendments. Informed consent was obtained from all subjects involved in the study.

**Table 1:** Demographic characteristics of the subjects.

PET tracer	Sex	Age (year)	Height (cm)	Weight (kg)	Injected dose (MBq)
^18^F-T807	Male	56.0 ± 3.6	170.3 ± 11.6	71.0 ± 14.5	246.3 ± 20.6
	Female	55.0 ± 3.5	160.0 ± 4.0	57.3 ± 9.5	278.7 ± 32.0
^18^F-Mefway	Male	29.0 ± 1.0	174.7 ± 3.5	70.7 ± 5.1	256.7 ± 26.6
	Female	39.0 ± 6.2	160.0 ± 5.0	52.7 ± 4.2	195.3 ± 14.6

*Enrolled subjects for each PET tracer are consisted of 6 subjects, 3 men and 3 women.

### PET scans

After administering ^18^F-T807 or ^18^F-Mefway, whole-body PET/CT images were obtained for all the subjects (Fig. 1). These whole-body PET images were obtained in eight contiguous segments from the vertex of the skull to the middle of the thigh using a Biograph 40 True Point system (Siemens Medical Solutions, Erlangen, Germany). The acquired time intervals were 0–8 min, 12–20 min, 24–32 min, 40–48 min, 60–68 min, 84–92 min, and 112–120 min for ^18^F-T807 (233–310 MBq) and 0–16 min, 20–36 min, 40–56 min, 60– 84 min, and 90–114 min for ^18^F-Mefway (182–274 MBq). Between each time interval, a rest period of 4 min was provided outside the gantry. To correct attenuation and scatter, as well as to obtain anatomic information, low-dose CT scans (35 mAs, 120 keV, 512 × 512 × 552 matrix, 3 mm slice thickness, 0.6 s rotation time) were performed. All emission data were reconstructed using ordered subset expectation maximization with 16 iterations.

### Image analysis

Ten volumes of interest (VOIs) were manually drawn on each coronal slice of the summed PET images with the assistance of the corresponding CT images. The brain, heart, lungs, liver, kidneys, gallbladder, small intestine, and urinary bladder were used as source organs. The most visible frame was chosen to draw the region and generate a VOI map. The VOI map was applied to dynamic PET to generate TACs. MATLAB and PMOD software were used for the TAC derivation.

Decay-uncorrected TACs and residence times for the major organs were derived by in-house software written in MATLAB (MathWorks, version 1.1, Vanderbilt University, USA), which we refer to as the conventional method. In addition, using the same PET data, we obtained the TACs and residence times using the Residence Times model, a dedicated analysis tool in PMOD software (version 3.7. PMOD Technologies). The residence times obtained using each method were compared. Thereafter, we input the residence time values obtained by each analysis method into OLINDA/EXM software (version 1.1, Vanderbilt University, USA), and then compared the absorbed dose and effective dose of the two radiotracers.

### Residence time and radiation dose calculation

#### Conventional method

The cumulative activity was calculated from the AUC of the TACs using an in-house software written in MATLAB (MathWorks). The trapezoidal method was applied to calculate the AUC for each organ, and the AUC from the last image to infinity was determined by the physical decay of ^18^F. The residence time, that is, the number of decays per injected activity, was derived by dividing the AUC by the total injected dose. To determine the residence time for the remainder of the body, the summed residence time for all the source organs was subtracted from the fixed theoretical value of 2.64 (= T_1/2_/ln2) for F-18^13^. OLINDA/EXM was used to derive human organ doses for adult males and females based on the residence times for each source organ. The absorbed dose (D) can be calculated by dividing the energy absorbed from the target organ by the mass of the target organ (m_t_), as follows:

$${\text{D}} = \frac{{\tilde{A}}}{{m_{t} }}\sum\limits_{i} {\emptyset_{i} \left( {rk \leftarrow r_{h} } \right)\Delta_{i} }$$

where ∅_i_ is the fraction of the energy emitted by the source organ (h) that is absorbed by the target organ (k), referred to as the absorbed fraction. A ~represents the cumulated activity in the source organ and refers to the AUC of the TAC. ∆_i_ represents the equilibrium absorbed dose constant, i.e., the energy emitted per unit of cumulated activity.

The effective dose (E) represents the overall risk to the whole body, calculated by summing the absorbed doses of each organ multiplied by its tissue weighting factor:

$${\text{E}} = \mathop \sum \limits_{T} w_{T } \times D_{T } \times w_{R}$$

where w_T_ is the tissue weighting factor for organ T, D_T_ is the average absorbed dose in organ T, and w_R_ is the radiation weighting factor used to calculate equivalent dose for different types and energies of radiation.

#### PMOD analysis

**Figure 2:**
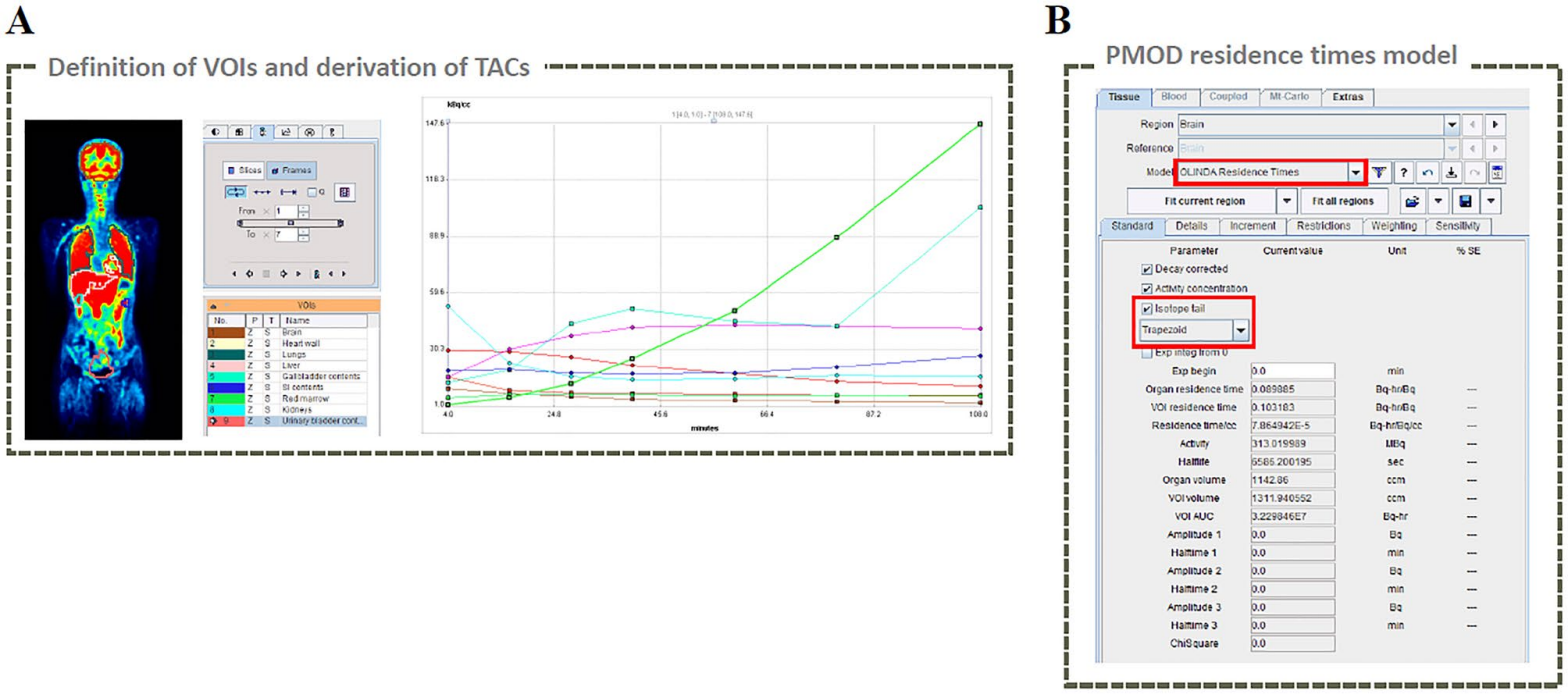
Calculation process of residence time for each organ using the PMOD Residence Times model. The process of defining VOI and deriving TAC in major organs is the same as that in the conventional method (**A**). Example window for calculating the residence time of the brain in the Residence Times model (**B**). Trapezoidal method chosen for calculating the AUC.

The Residence Times model in PMOD enables the automation of the residence time calculation based on TACs (Fig. 2A). The trapezoidal method was used to obtain the AUC for each organ. Because trapezoidal fitting is more suitable for delayed clearance form than exponential fitting, it is preferred in the case of rapid clearance. In addition, we checked the isotope toolbox to obtain the AUC from the last frame to infinity (Fig. 2B). These two simple checks allowed us to immediately derive the residence time of each organ without further calculations. OLINDA/EXM was used to calculate the absorbed dose and effective dose values, following the same procedure as for the conventional method. For both methods, we assumed that the bladder content volume was constant based on previous studies^14–16^.

## Results

**Figure 3:**
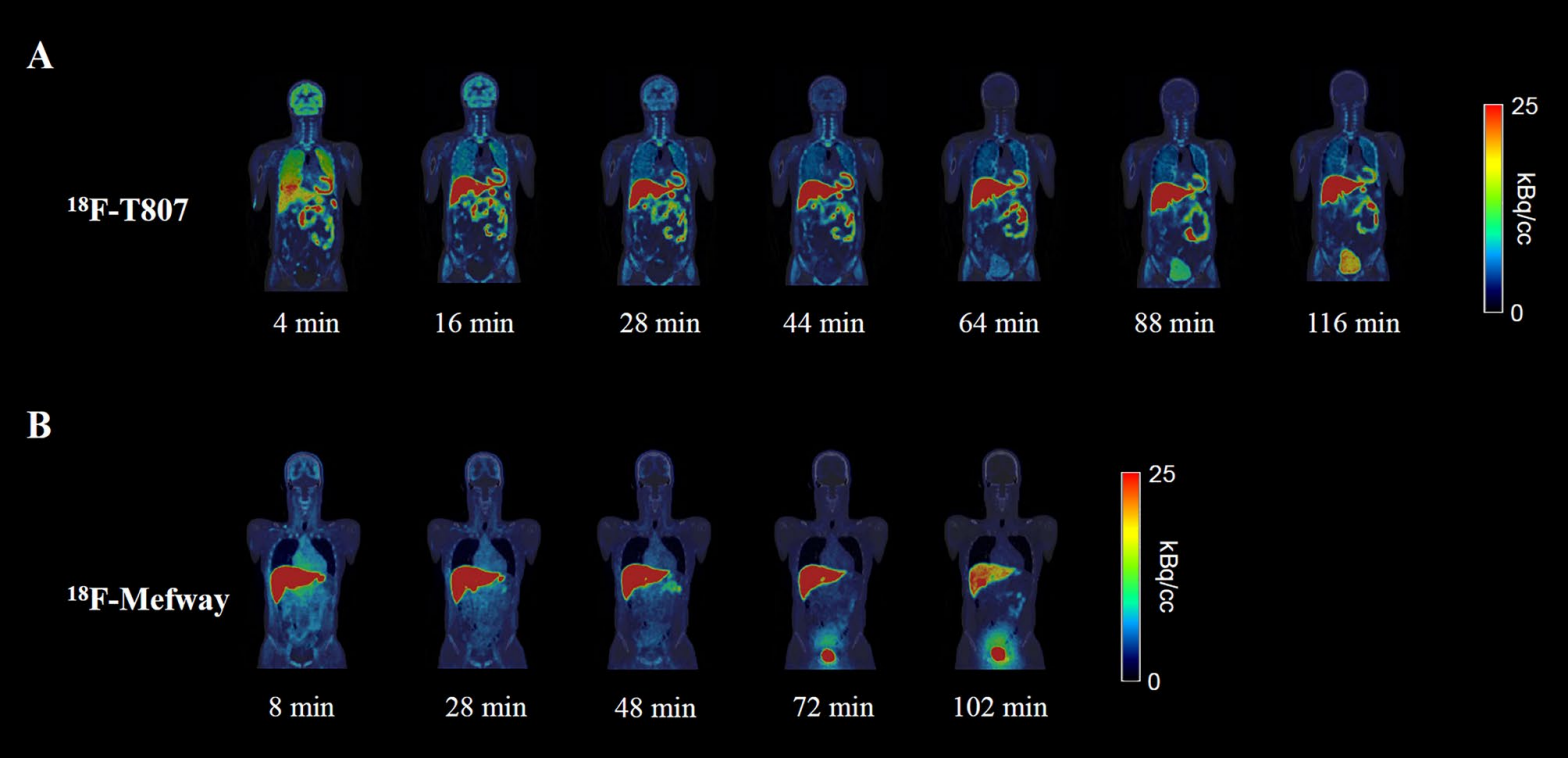
Whole-body images of a healthy subject at various time points after the injection of ^18^F-T807 (**A**) and ^18^F-Mefway (**B**).

Six healthy subjects participated in this study, with an average of 56 years old for ^18^F-T807 and 34 years old for ^18^F-Mefway. The amounts of radioactivity administered were 262.5 MBq and 226.0 MBq for ^18^F-T807 and ^18^F-Mefway, respectively (Table 1). Figure 3 shows the representative coronal PET/CT images of ^18^F-T807 (A) and ^18^F-Mefway (B) over time. ^18^F-T807 PET initially shows uptakes in the lungs, heart, intestine, and brain, and is maintained in the liver, gallbladder, and intestine in the later stages. In the case of ^18^F-Mefway, radioactivity accumulates and is rapidly washed out from the liver, kidneys, and brain, whereas the uptake in the urinary bladder increases over time.

Table 2 summarizes the average residence times of the source organs and the percentage differences calculated using the conventional method and PMOD for the six healthy subjects. For ^18^F-T807, the error rate between the two methods in calculating the average residence time decreases in the order of the urinary bladder (80%), gallbladder (37.9%), kidney (27.9%), heart wall (20.8%), brain (18.4%), liver (13.3%), lungs (10.4%), and small intestine (4.4%). In case of ^18^F-Mefway, the lungs show the largest difference of 18.1%, followed by the liver/ brain (17.5%), heart wall (16.4%), small intestine (12.8%), kidney (12.0%), and gallbladder (1.1%).

**Table 2:** A comparison of residence times from major source organs for ^18^F-T807 and ^18^F-Mefway.

Organs	^18^F-T807				^18^F-Mefway			
	Male (n = 3)		Female (n = 3)		Male (n = 3)		Female (n = 3)	
	Conventional	PMOD	CONVENTIONAL	PMOD	Conventional	PMOD	Conventional	PMOD
Brain	0.052 ± 0.005	0.044 ± 0.002	0.061 ± 0.023	0.048 ± 0.026	0.026 ± 0.005	0.021 ± 0.004	0.035 ± 0.002	0.029 ± 0.001
Heart wall	0.015 ± 0.005	0.011 ± 0.005	0.022 ± 0.001	0.018 ± 0.001	0.002 ± 0.001	0.001 ± 0.000	0.005 ± 0.002	0.004 ± 0.002
Lungs	0.191 ± 0.027	0.176 ± 0.031	0.227 ± 0.017	0.199 ± 0.017	0.048 ± 0.012	0.040 ± 0.012	0.074 ± 0.009	0.059 ± 0.008
Liver	0.518 ± 0.075	0.465 ± 0.093	0.587 ± 0.026	0.492 ± 0.007	0.398 ± 0.083	0.318 ± 0.069	0.442 ± 0.094	0.375 ± 0.118
Gallbladder wall	0.001 ± 0.001	0.001 ± 0.001	0.020 ± 0.012	0.034 ± 0.016	0.000 ± 0.000	0.000 ± 0.000	0.000 ± 0.000	0.000 ± 0.000
Small intestine	0.130 ± 0.019	0.124 ± 0.006	0.070 ± 0.015	0.067 ± 0.016	0.008 ± 0.003	0.007 ± 0.003	0.006 ± 0.001	0.005 ± 0.001
Kidneys	0.027 ± 0.016	0.040 ± 0.009	0.049 ± 0.018	0.043 ± 0.023	0.070 ± 0.017	0.060 ± 0.012	0.037 ± 0.006	0.034 ± 0.006
Urinary bladder wall	0.114 ± 0.089	0.258 ± 0.239	0.065 ± 0.035	0.087 ± 0.035	0.853 ± 0.014	0.877 ± 0.024	0.790 ± 0.163	0.834 ± 0.145
Reminder of body	1.390 ± 0.342	1.482 ± 0.387	1.410 ± 0.015	1.419 ± 0.052	1.179 ± 0.086	1.307 ± 0.040	1.186 ± 0.070	1.310 ± 0.108

*Data are presented as the mean ± the SD.

Table 3 summarizes the absorbed and effective doses of ^18^F-T807 and ^18^F-Mefway to the source organs. The absorbed dose for ^18^F-T807 is the highest in the liver and decreases in the order of the lungs, gallbladder, and urinary bladder in both analysis methods. The difference in the absorbed doses between both methods is large for the urinary bladder wall (49.7%), followed by the thyroid (31.8%) and heart wall (28.4%), and is the smallest in the liver (5.9%). In case of ^18^F-Mefway, the absorbed dose decreases in the order of the urinary bladder, liver, and kidney for both methods. The difference in the absorbed doses between both methods is large for the liver (16.0%), brain (11.8%), and kidneys (10.1%), and is smallest in the pancreas (0.9%). These results indicated that ^18^F-T807 is primarily excreted through the hepatobiliary system whereas, ^18^F-Mefway passes through the renal excretion system.

**Table 3:** Comparison of absorbed dose in the source organs between conventional method and PMOD.

Organs	^18^F-T807				^18^F-Mefway			
	Male (n = 3)		Female (n = 3)		Male (n = 3)		Female (n = 3)	
	Conventional	PMOD	Conventional	PMOD	Conventional	PMOD	Conventional	PMOD
Adrenals	16.3 ± 0.3	13.0 ± 4.2	21.3 ± 0.3	17.1 ± 4.6	12.6 ± 0.6	12.2 ± 0.8	15.9 ± 1.7	15.6 ± 2.4
Brain	11.2 ± 1.2	9.1 ± 0.3	14.2 ± 4.5	11.5 ± 3.6	5.8 ± 0.9	5.1 ± 0.8	8.6 ± 0.2	7.6 ± 0.3
Breasts	8.9 ± 0.4	6.3 ± 3.4	10.8 ± 0.1	8.0 ± 4.0	6.0 ± 0.2	6.3 ± 0.2	8.0 ± 0.2	8.3 ± 0.9
Gallbladder wall	29.4 ± 12.6	18.7 ± 3.6	60.3 ± 20.5	65.5 ± 34.0	16.8 ± 1.4	15.9 ± 1.6	20.4 ± 2.4	19.7 ± 3.6
Lower large intestine wall	12.2 ± 0.7	11.1 ± 6.0	13.8 ± 0.6	10.7 ± 6.0	19.0 ± 0.3	20.0 ± 0.5	23.2 ± 2.4	24.8 ± 2.1
Small intestine	33.5 ± 8.3	35.8 ± 0.9	29.6 ± 3.6	26.9 ± 4.5	14.4 ± 0.5	14.7 ± 0.9	16.7 ± 0.7	17.3 ± 0.7
Stomach wall	12.2 ± 0.2	9.1 ± 4.6	15.1 ± 0.2	11.5 ± 5.3	9.2 ± 0.1	9.5 ± 0.3	11.8 ± 0.8	12.2 ± 1.3
Upper large intestine wall	15.7 ± 1.2	13.8 ± 4.2	17.8 ± 0.6	14.5 ± 5.4	12.5 ± 0.1	12.8 ± 0.5	15.8 ± 0.1	16.5 ± 1.0
Heart wall	20.1 ± 1.4	15.3 ± 2.0	29.1 ± 0.9	19.5 ± 9.8	7.4 ± 0.4	7.0 ± 0.6	12.5 ± 2.3	11.6 ± 2.4
Kidneys	26.2 ± 8.6	30.6 ± 5.7	43.6 ± 11.7	38.5 ± 10.6	49.5 ± 10.8	43.2 ± 7.7	33.6 ± 4.7	31.2 ± 4.9
Liver	61.6 ± 6.0	61.4 ± 7.8	100.8 ± 3.9	89.3 ± 6.2	52.3 ± 10.0	42.8 ± 8.5	75.4 ± 15.2	64.9 ± 19.2
Lungs	41.3 ± 7.9	35.2 ± 3.8	57.7 ± 3.6	50.9 ± 2.4	13.2 ± 1.9	11.8 ± 1.9	22.8 ± 2.5	19.6 ± 2.5
Muscle	9.9 ± 0.3	7.5 ± 4.3	11.7 ± 0.1	8.8 ± 4.7	9.7 ± 0.2	10.2 ± 0.3	12.0 ± 0.1	12.6 ± 0.7
Ovaries	–	–	14.8 ± 0.6	11.7 ± 5.9		–	22.9 ± 2.2	24.4 ± 1.9
Pancreas	15.3 ± 0.1	12.1 ± 4.6	20.0 ± 0.3	15.9 ± 5.3	11.9 ± 0.4	11.7 ± 0.6	15.1 ± 1.5	15.1 ± 2.2
Osteogenic cells	17.7 ± 0.8	13.4 ± 6.0	23.3 ± 1.5	17.8 ± 7.6	12.9 ± 0.7	13.5 ± 0.6	17.5 ± 0.5	18.3 ± 1.3
Skin	7.3 ± 0.3	5.1 ± 3.4	8.4 ± 0.1	6.1 ± 3.8	6.2 ± 0.2	6.6 ± 0.2	7.7 ± 0.2	8.2 ± 0.6
Spleen	11.2 ± 0.3	8.2 ± 4.5	14.0 ± 0.4	10.6 ± 5.2	8.9 ± 0.2	9.2 ± 0.2	11.0 ± 0.8	11.5 ± 1.1
Testes	8.4 ± 0.5	9.6 ± 0.5	–	–	13.6 ± 0.4	14.4 ± 0.3	–	–
Thymus	10.7 ± 0.6	7.4 ± 4.2	12.9 ± 0.1	9.5 ± 5.1	7.0 ± 0.4	7.4 ± 0.2	9.4 ± 0.7	9.9 ± 1.0
Thyroid	9.1 ± 0.5	5.9 ± 4.4	9.7 ± 0.1	6.8 ± 4.7	6.4 ± 0.4	6.9 ± 0.2	7.6 ± 0.4	8.3 ± 0.7
Urinary bladder wall	32.2 ± 13.4	61.2 ± 32.5	51.8 ± 23.1	56.7 ± 23.5	410 ± 6.0	421.3 ± 11.5	532.0 ± 107.8	562.3 ± 95.9
Uterus			15.4 ± 1.2	12.6 ± 6.3			36.73 ± 5.05	38.97 ± 4.44
Effective dose								
(μSv/MBq)	19.2 ± 0.4	21.1 ± 7.3	25.7 ± 0.8	22.1 ± 4.3	34.9 ± 0.4	35.2 ± 1.15	45.6 ± 4.9	46.8 ± 4.9

*Data are presented as the mean ± the SD. All values were represented in units of μSv/MBq.

The effective doses of ^18^F-T807 and ^18^F-Mefway were estimated as 22.47 and 40.23 μSv/MBq from the conventional method. These values correspond well with those from PMOD (21.47 and 40.98 μSv/MBq, respectively). For the mean effective dose, the error rates between the methods were 3.8% and 1.9% for ^18^F-T807 and ^18^F-Mefway, respectively. In terms of sex comparison, females showed 4.1% and 1.8% higher effective doses than males for both radiotracers.

## Discussion

**Figure 4:**
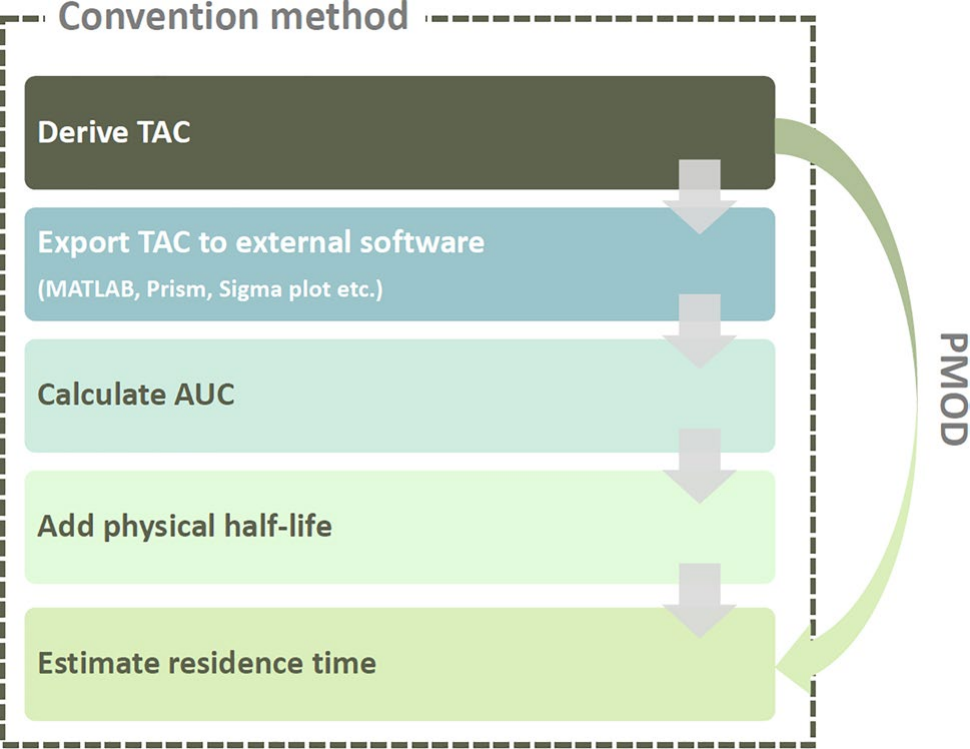
Comparison between conventional method and PMOD method for calculating the residence time.

The safety and effectiveness of radiotracers must be evaluated to conduct clinical trials, wherein internal radiation exposure is used as the evaluation parameter for safety. This internal radiation exposure can be calculated through dosimetry. Specifically, based on dynamic PET data for a specific radiotracer, the residence time for many organs can be obtained, and the effective dose and critical organs are determined based on this. To derive the residence time, conventionally, a formula designed by researchers was previously used; the process was complex and difficult, resulting in limited accessibility. However, if PMOD, an image analysis software, is used, this process is simplified (Fig. 4). In the present study, we observed that the difference in the effective doses between PMOD and conventional methods is below 4% using previously developed radiotracers (i.e., ^18^F-T807 and ^18^F-Mefway). The results of this study are expected to be clinically applicable as they can avoid complexity in the safety evaluation of radioactive drugs.

The effective dose is closely related to the safety of patients, and provides crucial information for go/no-go decision-making in clinical trials. To obtain an accurate estimate of this value, exhaustive data collection is essential. Therefore, a total-body PET scanner, capable of longer-duration data acquisition, can be superior to the PET scanner with a limited field of view (FOV). Recently, Hu et al., reported that an 8 h dynamic scan using a total-body PET scanner showed an increase in the cumulative activity in the liver by more than 18.4% compared to a short-duration PET (75 min) scan. Furthermore, they proved that the error rate in the residence time from long and short acquisition times for the brain, heart, kidney, liver, and lung ranged from 8 to 24%^17^. However, including our institution, most hospitals are typically equipped with limited FOV PET systems rather than total-body PET systems. In this case, having patients lie on a bed for a long period is challenging, and their movement could potentially degrade the quality of the images. Therefore, we evaluated the validity of the Residence Times model of PMOD in dosimetry analysis based on the most widely used PET imaging protocol, i.e., short-duration image acquisition using limited FOV PET systems.

The error rate in the effective doses for both radiotracers was below 4% between the methods. However, despite the high error rates in the residence times for the urinary bladder, ^18^F-T807 showed minimal differences in the effective dose. This is due to the physiological characteristics of ^18^F-T807, which exhibits hepatobiliary excretion. Therefore, ^18^F-T807 exhibits the pharmacokinetic characteristics of being excreted through the liver, gallbladder, and intestines. Importantly, the role of the urinary bladder in the distribution and elimination of ^18^F-T807 is negligible, and it is believed to have a minimal impact on the effective dose. Another hypothesis is that the tissue weighting factor for the urinary bladder is 0.04, which is not relatively high compared to that for other major organs (brain, 0.12; breast, 0.12; lung, 0.12; stomach, 0.12; kidney, 0.12; colon, 0.12). Therefore, the urinary bladder has a relatively higher resistance to radiation, and contributes lesser to the effective dose compared to other radiation-sensitive tissues. If the urinary bladder has the longest residence time, the effective dose may be reduced by consuming sufficient water before PET scan, e.g., in a ^18^F-FDG PET scan.

Sex is one of the important factors that can influence radiation sensitivity owing to physiological differences. The breast and reproductive tissues (e.g., uterus, ovaries) of females are generally more sensitive compared to those of males. Therefore, the internal distribution and excretion of radiation within females can be different from that in males, and the radiation exposure is generally higher. This is an important factor to consider during radiation therapy and diagnostic procedures. According to data from Nuclear Information and Resources Services (NIRS), females exhibit a 50% higher incidence of cancer compared to males who receive the same radiation dose, indicating that radiation may have a more significant impact on females^18^. Narendran et al. reported that radiation effects exhibit sex-specific differences, with females displaying higher radiosensitivity compared to males^19^. In the present study, we observed that the effective doses in females were relatively higher than in males, and this result was consistent with both, the conventional method and PMOD Residence Times model analysis. Consequently, internal radiation dose assessments can vary based on sex, and for diagnostic evaluations using ^18^F-T807 and ^18^F-Mefway, we recommend limiting long-term usage in females.

The present study has several limitations. First, our work is a retrospective study, and as such, it did not derive S values from individual participants. Second, despite the differences in residence times and absorbed doses between the two analytical methods, the study did not elucidate the reason or mechanism for the minimal difference in the effective dose. To address this, it may be necessary to conduct individualized S value analyses to examine various effective calculation factors. Finally, we did not control the subjects’ age, radiotracer injection dose, and degree of hydration before the PET scan.

## Conclusion

Evaluating the internal radiation dose for radiotracers from whole-body dynamic PET is important because it secures the safety of the drug. In this study, PMOD was used in the dosimetry process to evaluate the residence time more simply and easily than the conventional method. Although the results are not based on data from sufficient subjects, we observed that the difference of the effective doses for both methods was below 4%. In addition, there would be fewer deviations for PET tracers performing renal clearance than hepatobiliary excretion. These results are expected to be available to easily evaluate the internal exposure dose of the radioactive follower under development.

## Data Availability

The materials and datasets used and/or analyzed during the current study are available from the corresponding author upon reasonable request.
